# Characterization of *PCLO* Gene in Amazonian Native American Populations

**DOI:** 10.3390/genes13030499

**Published:** 2022-03-11

**Authors:** Amanda de Nazaré Cohen-Paes, Darlen Cardoso de Carvalho, Lucas Favacho Pastana, Elizabeth Ayres Fragoso Dobbin, Fabiano Cordeiro Moreira, Tatiane Piedade de Souza, Marianne Rodrigues Fernandes, Diana Feio da Veiga Borges Leal, Roberta Borges Andrade de Sá, Angélica Leite de Alcântara, João Farias Guerreiro, Ândrea Ribeiro-dos-Santos, Sidney Emanuel Batista dos Santos, Paulo Pimentel de Assumpção, Ney Pereira Carneiro dos Santos

**Affiliations:** 1 Núcleo de Pesquisa em Oncologia, Federal University of Pará, Belém 66073-000, Brazil; acohencastro@gmail.com (A.d.N.C.-P.); darlen.c.carvalho@gmail.com (D.C.d.C.); lucas.favacho.celular@gmail.com (L.F.P.); elizabethdobbin7@gmail.com (E.A.F.D.); fabiano.ufpa@gmail.com (F.C.M.); dianafeio@hotmail.com (D.F.d.V.B.L.); robertaborgesandrade@gmail.com (R.B.A.d.S.); angelica.alcantara99@gmail.com (A.L.d.A.); akelyufpa@gmail.com (Â.R.-d.-S.); sidneysantos@ufpa.br (S.E.B.d.S.); assumpcaopp@gmail.com (P.P.d.A.); npcsantos@yahoo.com.br (N.P.C.d.S.); 2 Laboratório de Genética Humana e Médica, Federal University of Pará, Belém 66073-000, Brazil; tatiane.piedadeps@gmail.com (T.P.d.S.); joao.guerreiro53@gmail.com (J.F.G.)

**Keywords:** *PCLO*, Native American, indigenous populations, Brazil, genetic variants

## Abstract

Genetic variations in *PCLO* have been associated with different pathologies in global literature, but there are no data regarding this gene in Native American populations. The Amazonian Native American populations have lower genetic diversity and are more different from other continental groups. We investigated 18 genetic variants in the *PCLO* gene in Amazonian indigenous and compared our results with the ones found in global populations, which were publicly available in the 1000 Genomes Project, gnmAD and ABraOM databases. The results demonstrated that the variants of the *PCLO*, especially rs17156844, rs550369696, rs61741659 and rs2877, have a significantly higher frequency in Amerindian populations in comparison with other continental populations. These data outline the singular genetic profile of the Native American population from the Brazilian Amazon region.

## 1. Introduction

The *PCLO* gene on chromosome 7q21.11 (GRCh38) encodes a presynaptic cytomatrix protein called piccolo (piccolo presynaptic cytomatrix protein), which is involved in the establishment of active synaptic zones and in synaptic vesicle trafficking [1,2,3]. *PCLO* is studied in neuroscience, as it plays a role in modulating monoaminergic transmission, e.g., transmission of serotonin (5-HT), adrenaline, noradrenaline and dopamine [4,5]. Piccolo is highly expressed in brain and adrenal glands, as well as adipose tissue, gallbladder, pancreas, stomach, thyroid and esophagus [6,7,8]. The unstable gene expression and genetic variation caused by mutations in *PCLO* have been associated with different pathologies in global literature, such as major depressive disorder [9,10], bipolar disorder [11], cancer [5] and diabetes [12,13]. There were few studies investigating the genetic profile of *PCLO* in general populations, but there are no data regarding this gene in Amerindian population. Information present in clinical databases—such as ClinVar, from National Center for Biotechnology Information (NCBI; accessed on 13 January 2022, at: https://www.ncbi.nlm.nih.gov/clinvar/) and Brazilian Initiative on Precision Medicine (https://bipmed.org/, accessed on 13 January 2022)—regarding this gene are also rare.

In the latest census in 2010, the Brazilian government estimated the Native Amerindian population in approximately 897,000 individuals with 305 ethnicities. This population has been reported in all states in Brazil; however, they are highly concentrated in the north of the country, within the Amazon region [14]. Great efforts have been made towards understanding the social and cultural pattern of this group; however, little research has been done on their genetic profile. In addition, genetic studies have indicated that highly mixed populations—particularly those with high Amerindian ancestry—are more susceptible to certain diseases, such as cancer and tuberculosis [15,16,17,18]. The investigation of genetic variants Single Nucleotide Polymorphisms (SNPs) and insertion/deletion polymorphism (INDELs) enable the discovery and the characterization of new molecular markers in this population, and also validates the knowledge from previous studies in other world populations. These genetic variations in *PCLO* gene can be compared intra- and interpopulation. Additionally, the molecular markers may have clinical applications, working as diagnosis and treatment tools for the Amerindian population and mixed populations with strong Amerindian ancestry, as in Brazil [17].

Therefore, this study aimed to characterize the *PCLO* molecular profile in Amerindian populations from the Brazilian Amazon, and to compare these findings with data from the general Brazilian population described in ABraOM, as well as with the five continental populations available in the 1000 Genomes Project and Genome Aggregation Database (gnomAD).

## 2. Materials and Methods

### 2.1. Study Populations

The study was approved by the National Research Ethics Committee (CONEP; available at: http://conselho.saude.gov.br/comissoes-cns/conep/; accessed on 13 January 2022) and by the Research Ethics Committee of the Tropical Medicine Center of the Federal University of Pará (CAE: 20654313.6.0000.5172). All individuals and community leaders signed an informed consent form (TCLE).

The study population consisted of 63 Amerindians from the Brazilian Amazon in Northern Brazil. Amerindians represent 12 different Amazonian ethnic groups: Asurini do Xingu, Arara/Arara do Iriri, Araweté, Asurini do Tocantins, Awa-Guajá, Kayapó/Xikrin, Zo’é, Wajãpi, Karipuna, Phurere, Munduruku and Yudjá/Juruna. For statistical analysis, these individuals were grouped into a single sample named NAT (Native American Population).

The Amerindian population data were compared with representatives of five continental populations obtained from the 1000 Genomes Project, a public catalog of human variation and genotype data (available at: http://www.1000genomes.org; accessed on 13 January 2022). This sample included 661 individuals from Africa (AFR), 503 from Europe (EUR), 347 from the Americas (AMR), 504 from East Asia (EAS) and 489 from South Asia (SAS).

In order to use data with a larger sample, we also added to our analysis population data available in the Genome Aggregation Database (available at: https://gnomad.broadinstitute.org/), for the five continental populations investigated (AFR, EUR, AMR, EAS and SAS). Finally, the Native American population of the Amazon was also compared in our analyzes with the Brazilian population, which were obtained from the ABraOM database, (ABraOM, Sao Paulo, Sao Paulo; available at: https://abraom.ib.usp.br/) a repository containing genomic variants gathered by sequencing the complete exome of individuals from São Paulo, the largest city of Brazil, located in the Southwest Region. All databases were accessed on 13 January 2022 to extract genomic data.

### 2.2. Extraction of the DNA and Preparation of the Exome Library

DNA was extracted from a peripheral blood sample using the phenol-chloroform method described by Sambrook et al. [19]. The Nanodrop-8000 spectrophotometer (Thermo Fisher Scientific Inc., Wilmington, DE, USA) was used to quantify the genetic material and its integrity was evaluated by 2% agarose gel electrophoresis.

Libraries were prepared using the Nextera Rapid Capture Exome (Illumina^®^, San Diego, CA, USA) and SureSelect Human All Exon V6 (Agilent technologies, Santa Clara, CA, USA) kits, following the manufacturer’s recommendations. The sequencing reactions were performed on the NextSeq 500^®^ platform (Illumina^®^, San Diego, CA, USA) using the NextSeq 500 High-output v2 Kit 300 cycle kit (Illumina^®^, San Diego, CA, USA).

### 2.3. Bioinformatic Analysis

Reads in FASTQ format were analyzed for quality (FastQC v.0.11 http://www.bioinformatics.babraham.ac.uk/projects/fastqc/, accessed on 25 June 2021) and filtered to eliminate low quality reads (fastx_tools v.0.13 http://hannonlab.cshl.edu/fastx_toolkit/; accessed on 25 June 2021). Then, the sequences were aligned with the reference genome (GRCh38) using the BWA v.0.7 tool (http://bio-bwa.sourceforge.net/; accessed on 25 June 2021).

After alignment to the reference, the generated file was indexed and sorted (SAMtools v.1.2—http://sourceforge.net/projects/samtools/; accessed on 25 June 2021). Subsequently, the alignment had to be processed to remove duplicate readings (Picard Tools v.1.129—http://broadinstitute.github.io/picard/; accessed on 25 June 2021), mapping quality recalibration and local realignment (GATK v.3.2—https://www.broadinstitute.org/gatk/; accessed on 25 June 2021). Finally, the result was processed in search of variants (GATK v.3.2, United States; https://gatk.broadinstitute.org/hc/en-us) of the reference genome. The allelic variants were annotated in the ViVa^®^ (Viewer of Variants, Natal, RN, Brazil) software. Markers in *PCLO* gene were also selected in coverage, where the readout should be high coverage, with a minimum of 10 reads (fastx_tools v.0.13-http://hannonlab.cshl.edu/fastx_toolkit/; accessed on 25 June 2021).

### 2.4. Statistical Analyses

All statistical analyzes were performed using the R Studio v.3.5.1 program (R Foundation for Statistical Computing, Vienna, Austria), including the Discriminant Analysis of Principal Components (DAPC). Significant differences in allele frequencies between populations were analyzed by Fisher’s exact test. The False Discovery Rate (FDR) proposed by [20] was used to correct the multiple analyses. Results were considered statistically significant when the *p*-value was less than 0.05 (*p* ≤ 0.05).

## 3. Results

A total of 18 variants were identified in the Amazonian NAT, with 16 SNVs (Single Nucleotide Variants) and two INDELs (Insertion–Deletion). Based on the location and the type of the genetic modifications, 12 variants of *PCLO* were classified as moderate impact, two variants as modifier impact and four as low impact variants. Table 1 summarized characteristics of the markers investigated in NAT and in the populations described in the 1000 Genomes Project, gnomAD and ABraOM databases.

Table 2 shows the detailed allelic frequencies of the investigated markers in *PCLO*. Frequency data of each variant in the five continental populations were taken from the 1000 Genomes project database and the frequency of markers in the Brazilian population was taken from ABraOM, so that we could compare them to the frequencies of NAT populations. Six of the eighteen variants were not found in NAT populations. The rs550369696 variant was found only in NAT population. The rs762371134 have no frequency data in the 1000 Genomes Project, so the frequency data presented in this database are the result of the gnomAD analysis. Thus, we represented their frequencies by “ND”, which means “no data” for this variant.

Table 3 shows the detailed frequencies of the investigated markers in the *PCLO* gene according to the data described in gnomAD browser. Table 3 also presents the frequencies for the same markers in the Brazilian population, which is described in the ABraOM database.

For the American population of the 1000 Genomes project, three of the investigated variants showed significant difference from the NAT population (rs17156844, rs550369696 and rs2877) (Table 4). We would like to highlight that SNV rs550369696 is distributed differentially in the NAT population when compared to all continental populations investigated. Likewise, when we highlight the SNV rs17156844, the results show that, for the six populations investigated, five statistically differ from the NAT population, including the ABraOM population. For rs2877, the number of markers decreases to four, with three continental populations (AMR, AFR and EUR) added to the ABraOM population. When we compare NAT-AFR, we can see that the population in which the *PCLO* gene markers differed the most was the African one, with 10 of the 18 having frequencies that are notably higher in Native American populations (rs17156844, rs2715150, rs550369696, rs2877, rs976714, rs2522833, rs10261848, rs28680905, rs17148149 and rs12668093).

When we analyzed the results from gnomAD, we noticed some differences with the data presented above for the 1000 Genomes Project (Table 5). Only the AMR (*p*-Value = 0.0021) and EUR (*p*-Value = < 0.0001) populations were statistically different from the NAT population regarding the SNV rs17156844. For the marker rs550369696, the Native American population did not have higher frequencies when compared to any of the continental populations or the ABraOM. However, the NAT-AFR analysis remains significant, with six (rs2715150, rs2522833, rs10261848, rs28680905 and rs12668093) of the eighteen investigated variants being more frequent in NAT than in the African population, of which all obtained the same result in the analysis of the NAT-AFR in 1000 Genomes. In the results compared to the ABraOM population, four markers (rs2715150, rs61741659, rs762371134 and rs2522833) showed a higher frequency of distribution in the Amerindian population, and with the exception of rs762371134, all of which obtained the same results in the above-mentioned analysis.

The comparative analysis of these two databases allows a more accurate visualization of the genomic data of the investigated population, as the databases use different sample numbers for each investigated continental population. Thus, the differences and similarities of each database are within the expected range.

**Figure 1:**
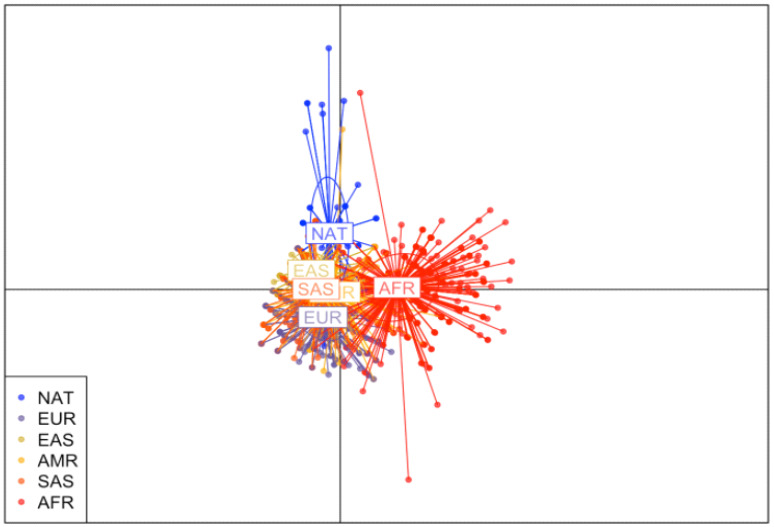
Discriminant Analysis of Principal Components of *PCLO* gene in Native American populations and individuals Europe, East Asia, Americas, South Asia and Africa. Native American population (NAT), African population (AFR), European population (EUR), American population (AMR), East Asian population (EAS) and South Asian population (SAS).

Discriminant Analysis of Principal Components (DAPC) Scatterplot is presented in Figure 1. The analysis divided the studied populations into well-defined clusters according to the genetic structure in the *PCLO* gene, modulated in the five continental populations described in the 1000 Genomes Project database and in the gnomAD database. It demonstrated a more significant distance between the population of interest (NAT) and the African population, while showing a greater proximity between the NAT population and the EAS and SAS populations.

## 4. Discussion

*PCLO* encodes Piccolo, a protein that builds the presynaptic cytoskeletal matrix, which is believed to be involved in modulating neurotransmitters’ release [21,22]. Studies demonstrated that loss of Piccolo and Bassoon (another component of the presynaptic zone) leads to aberrant degradation of several presynaptic proteins, culminating in the degeneration of synapses [6]. Genetic variations in the *PCLO* gene in humans can lead not only to synaptic dysfunction, but also to loss of brain and cerebellar volume, suggesting severe neuronal loss [23]. Studies on this gene demonstrate that the imbalance in its expression can modulate the development of different diseases [5,13,24]. 

To date, few genetic screening investigations have been carried out in Amerindian populations, especially in the Brazilian Amazon ones; thus, this group is epidemiologically and genetically understudied [14]. Traditional Latin America populations are a complex study group due to their human history of admixed, which gives them high levels of population genetic diversity [25]. The study by Ribeiro-dos-Santos [26] sequenced the genome of an individual from a South American tribe and identified more than 60,000 new genetic variants, among them, specific variants of the South America native populations. These results demonstrated the need for a deeper understanding of the genetic variability of South American Amerindian populations [26]. Other recent investigations have shown that Brazilian Amerindians have a unique genetic profile [17,27,28,29]. Studies have increasingly demonstrated that next-generation sequencing methodologies are important to guide us to new and rare variants. It is also known that the frequencies’ fluctuation of polymorphisms in different ethnic groups can lead to important consequences, such as an association with complex diseases, which justifies the relevance of studying the molecular epidemiology of a population [30].

This is the first study to investigate the *PCLO* gene in Amazonian Amerindians, a population not described in the largest available databases on human genetic variability, the 1000 Genomes Project and gnomAD. Brazilian efforts to identify genetic variants, such as ABraOM, show the importance of not only investigating Brazilian population, but also the various indigenous ethnic groups spread across all country. Our results showed that, although many investigated variants have a similar frequency distribution between continental populations and the population described in ABraOM, at least 10 variants differed significantly between Native American and African populations in the analysis of 1000 Genomes. When looking at the NAT-AFR data in gnomAD, the rs2715150, rs2522833, rs10261848, rs28680905 and rs12668093 populations showed the same result as the other analysis for these populations, corroborating data regarding the genetic variability between Native American populations and other continental populations. Likewise, the DAPC analysis of *PCLO* showed that the NAT and AFR populations are the most genetically distinct, confirming what is known about history of human populations, which indicates that the Amerindian and African groups represent the extremes of the evolutionary process [31].

We also found in both analyzes that the frequencies of the variants rs17156844 (*p*-Value 1000 Genomes = 0.0060; *p*-Value gnomAD = 0.0021) and rs2877 (*p*-Value 1000 Genomes = 0.0013; *p*-Value gnomAD = 0.0232) are distributed differently in the indigenous population, showing high frequency in the Amazonian Native compared with the American population. These data agree with the finding by Wang and collaborators [32], who analyzed the genetic diversity and the populational structure of Native Americans from Central, North and South America, and then divided the genotypes of the individuals using a model of six clusters, which corresponded mainly to isolated populations of Ache and Suruí in South America. The authors concluded that South American indigenous populations are even more genetically isolated from other American populations [32]. In addition, when comparing regions within the Americas, the highest FST value was observed in eastern South America, demonstrating an overall lower level of Native American genetic variability in that region [32].

All populations included in the study had lower allele frequencies than the NAT population in at least one *PCLO* gene marker in both analyses. NAT-ABraOM data infer that the variants rs17156844 (*p*-Value = < 0.0001), rs2715150 (1000 Genomes *p*-Value = 0.0500; gnomAD *p*-Value = 0.05006), rs61741659 (*p*-Value = 0.0250; gnomAD *p*-Value = 0.02507), rs2877 (*p*-Value = < 0.0001) and rs2522833 (100 Genomes *p*-Value = 0.0500; gnomAD *p*-Value = 0.0500) differed significantly between these populations. Our result endorses the observation of Santos et al. [33] and Amador et al. [34], who investigated allelic and genotypic proportions of the Brazilian population for different purposes, but both showed that Brazilian individuals have different proportions of ancestral contribution in each country region. The Southwest Region of Brazil (where the ABraOM samples come from) has a lower ancestral contribution from Native American populations, and therefore, they are expected to be more different from NAT individuals than populations from the North Region of the country, for example [33,34]. Thus, this result highlights that, although the Brazilian population has significant proportions of NAT genes throughout its territory, its genetic profile may differ intrapopulationally, and therefore, it is important to trace its genome to discover their particularities.

Finally, the knowledge of the different patterns of genetic diversity in human populations is important in many health and genetic areas, as it can be used, for example, to investigate and validate new insights in the study of complex diseases, thus understanding the predisposition, diagnosis, prognosis and therapies for indigenous populations, and for populations with a high level of admixed, such as the Brazilian one. Therefore, we aimed to collaborate in the creation of public policies that help to optimize the quality of life of indigenous populations. Furthermore, the knowledge produced is also basis for inferences about human evolutionary history [35,36].

## 5. Conclusions

The results showed that *PCLO* variants have a significantly higher frequency in Native American populations, especially for rs17156844, rs550369696, rs61741659 and rs2877, than in other continental populations, as well as in population of Southwest Brazil. The data generated in the present study contributed to the understanding of the genetic influence of *PCLO* in a poorly studied group, in addition to providing important subsidies for future association studies, which aim to identify individuals more susceptible to pathologies due to genetic alterations of *PCLO* in Native American populations, as well as in other populations with a high degree of admixed with these groups. This is the first study to perform the *PCLO* exome in NAT populations from the Brazilian Amazon region; thus, we hope that these data may help in the establishment of future public policies for this population.

## Figures and Tables

**Table 1:** Characteristics of the genetic markers of *PCLO* investigated in African, American, East Asian, European, South Asian, Brazilians from Sao Paulo and Native American populations.

Genomic Position	SNP Identification (SNP ID)	Nucleotide Change	Impact Predicted by SNPeff	Variant Type
7:82915671	rs17156844	A > G	Low	SNV
7:82826579	rs2715150	A > G	Modifier	SNV
7:83135105	rs550369696	T > C	Low	SNV
7:83155781	rs61741659	T > C	Moderate	SNV
7:83135109	rs2877	C > G	Moderate	SNV
7:83155515	rs762371134	T > GTG	Moderate	INDEL
7:82952543	rs976714	C > T	Moderate	SNV
7:82952172	rs10630259	T > TTCA	Moderate	INDEL
7:82824392	rs2522833	A > C	Moderate	SNV
7:82953530	rs10954696	C > T	Moderate	SNV
7:82952942	rs10261848	T > G	Moderate	SNV
7:82951497	rs76988622	T > C	Low	SNV
7:82966426	rs28680905	T > C	Moderate	SNV
7:82902684	rs182078347	T > C	Moderate	SNV
7:82966518	rs6963292	G > A	Modifier	SNV
7:82953100	rs377241009	G > T	Moderate	SNV
7:82953964	rs17148149	C > T	Moderate	SNV
7:82805717	rs12668093	C > T	Low	SNV

**Table 2:** Allele frequencies of the variants of *PCLO* investigated in Native American population (NAT), in the Brazilian population of Sao Paulo (ABraOM) and in African (AFR), European (EUR), American (AMR), East Asian (EAS) and South Asian (SAS) from the 1000 Genomes Project.

SNP ID	AMR	AFR	EAS	EUR	SAS	NAT	ABraOM
rs17156844	0.499	0.502	0.547	0.203	0.380	0.758	0.332
rs2715150	0.416	0.042	0.687	0.432	0.556	0.556	0.339
rs550369696	0.001	0.000	0.000	0.000	0.000	0.062	ND
rs61741659	0.147	0.123	0.067	0.266	0.172	0.039	0.197
rs2877	0.781	0.694	0.993	0.684	0.920	0.984	0.684
rs762371134	ND	ND	ND	ND	ND	0.500	0.082
rs976714	0.389	0.103	0.440	0.361	0.399	0.453	0.305
rs10630259	0.994	1.000	0.996	0.987	0.991	1.000	0.993
rs2522833	0.416	0.042	0.687	0.432	0.556	0.565	0.339
rs10954696	0.389	0.102	0.442	0.361	0.399	0.431	0.305
rs10261848	0.019	0.213	0.000	0.000	0.008	0.009	0.037
rs76988622	0.004	0.032	0.000	0.000	0.000	0.000	0.014
rs28680905	0.013	0.179	0.000	0.000	0.000	0.000	0.034
rs182078347	0.013	0.000	0.000	0.000	0.000	0.000	0.003
rs6963292	0.020	0.279	0.000	0.000	0.008	0.000	0.048
rs377241009	0.000	0.000	0.000	0.000	0.000	0.000	ND
rs17148149	0.020	0.280	0.000	0.000	0.008	0.000	0.048
rs12668093	0.097	0.338	0.284	0.141	0.109	0.083	0.172

**Table 3:** Allele frequencies of the variants of *PCLO* investigated in Native American population (NAT), in the Brazilian population of Sao Paulo (ABraOM) and in African (AFR), European (EUR), American (AMR), East Asian (EAS) and South Asian (SAS) from the gnomAD Database.

SNP ID	AMR	AFR	EAS	EUR	SAS	NAT	ABraOM
rs17156844	0.499	0.431	0.604	0.211	0.322	0.758	0.332
rs2715150	0.449	0.093	0.704	0.423	0.576	0.556	0.339
rs550369696	0.000	0.000	0.000	0.000	0.000	0.062	ND
rs61741659	0.129	0.138	0.053	0.235	0.192	0.039	0.197
rs2877	0.837	0.708	0.993	0.688	0.892	0.984	0.684
rs762371134	0.196	0.010	0.215	0.031	0.115	0.500	0.082
rs976714	0.412	0.127	0.497	0.359	0.391	0.453	0.305
rs10630259	0.995	0.998	1.000	0.982	0.987	1.000	0.993
rs2522833	0.453	0.096	0.706	0.426	0.582	0.565	0.339
rs10954696	0.411	0.126	0.499	0.359	0.392	0.431	0.305
rs10261848	0.009	0.205	0.000	0.000	0.008	0.009	0.037
rs76988622	0.009	0.031	0.000	0.000	0.000	0.000	0.014
rs28680905	0.007	0.174	0.000	0.000	0.000	0.000	0.034
rs182078347	0.009	0.000	0.000	0.000	0.000	0.000	0.003
rs6963292	0.009	0.255	0.000	0.000	0.008	0.000	0.048
rs377241009	0.000	0.000	0.000	0.000	0.000	0.000	ND
rs17148149	0.009	0.258	0.000	0.000	0.008	0.000	0.048
rs12668093	0.110	0.307	0.289	0.144	0.144	0.083	0.172

**Table 4:** Pairwise comparison (*p*-value) of allelic frequencies in Amerindians and with each one of the five continental populations from 1000 Genomes and the Brazilian population from ABraOM for each *PCLO* gene variant investigated.

SNP ID	NAT vs. AMR	NAT vs. AFR	NAT vs. EAS	NAT vs. EUR	NAT vs. SAS	NAT vs. ABraOM
rs17156844	0.0060	0.0057	0.0689	<0.0001	<0.0001	<0.0001
rs2715150	1	<0.0001	1	1	1	0.0500
rs550369696	0.0488	0.0047	0.0133	0.0134	0.0148	ND
rs61741659	0.7027	1	1	0.0003	0.1424	0.0250
rs2877	0.0013	0.00012	1	<0.0001	1	<0.0001
rs762371134	ND	ND	ND	ND	ND	ND
rs976714	1	<0.0001	1	1	1	1
rs10630259	1	1	1	1	1	1
rs2522833	1	<0.0001	1	1	1	0.0500
rs10954696	1	1	1	1	1	1
rs10261848	1	0.0016	1	1	1	1
rs76988622	1	1	1	1	1	1
rs28680905	1	0.0008	1	1	1	1
rs182078347	1	1	1	1	1	1
rs6963292	1	1	1	1	1	1
rs377241009	1	1	1	1	1	ND
rs17148149	1	<0.0001	1	1	1	1
rs12668093	1	0.0003	0.0195	1	1	1

**Table 5:** Pairwise comparison (*p*-value) of allelic frequencies in Amerindians and with each one of the five continental populations from the gnomAD database for each *PCLO* gene variant investigated.

SNP ID	NAT vs. AMR	NAT vs. AFR	NAT vs. EAS	NAT vs. EUR	NAT vs. SAS	NAT vs. ABraOM
rs17156844	0.0021	6.86 × 10^8^	0.8747	<0.0001	4.26 × 10^3^	3.19 × 10^5^
rs2715150	1	0.0001	1	1	1	0.05006
rs550369696	1.51 × 10^6^	3.05 × 10^7^	1.87 × 10^6^	1.30 × 10^4^	2.46 × 10^6^	ND
rs61741659	1	0.8509	1	0.0010	0.02913	0.02507
rs2877	0.0232	1.18 × 10^8^	1	1.75 × 10^4^	1	3.26 × 10^7^
rs762371134	4.83 × 10^8^	<0.0001	6.02 × 10^9^	<0.0001	4.02 × 10^2^	1.32 × 10^1^
rs976714	1	1.69 × 10^6^	1	1	1	1
rs10630259	1	1	1	1	1	1
rs2522833	1	<0.0001	1	1	1	0.0500
rs10954696	1	7.79 × 10^6^	1	1	1	1
rs10261848	1	0.0012	0.62196	0.10114	1	1
rs76988622	1	1	1	1	1	1
rs28680905	1	0.0006	1	1	1	1
rs182078347	1	1	1	1	1	1
rs6963292	1	1.26 × 10^8^	1	1	1	1
rs377241009	1	1	1	1	1	ND
rs17148149	1	6.99 × 10^7^	1	1	1	1
rs12668093	1	0.0014	0.0064	1	1	1

## Data Availability

Not applicable.

## References

[1] Fenster S.D., Kessels M.M., Qualmann B., Chung W.J., Nash J., Gundelfinger E.D., Garner C.C. (2003). Interactions between Piccolo and the actin/dynamin-binding protein Abp1 link vesicle endocytosis to presynaptic active zones. J. Biol. Chem..

[2] Limbach C., Laue M.M., Wang X., Hu B., Thiede N., Hultqvist G., Kilimanne M.W. (2011). Molecular in situ topology of Aczonin/Piccolo and associated proteins at the mammalian neurotransmitter release site. Proc. Natl. Acad. Sci. USA.

[3] Minelli A., Scassellati C., Cloninger C.R., Tessari E., Bortolomasi M., Bonvicini C., Giacopuzzi M., Frisoni G.B., Gennarelli M. (2012). PCLO gene: Its role in vulnerability to major depressive disorder. J. Affect. Disord..

[4] Schuhmacher A., Mo¨ssner R., Ho¨fels S., Pfeiffer U., Guttenthaler V., Wagner M., Schwab S.G., Maier W., Zobel A. (2011). PCLO rs2522833 modulates HPA system response to antidepressant treatment in major depressive disorder. Int. J. Neuropsychopharmacol..

[5] Zhang W., Hong R., Xue L., Ou Y., Liu X., Zhao Z., Xiao W., Dong D., Dong L., Fu M. (2017). Piccolo mediates EGFR signaling and acts as a prognostic biomarker in esophageal squamous cell carcinoma. Oncogene.

[6] Waites C.L., Leal-Ortiz S.A., Okerlund N., Dalke H., Fejtova A., Altrock W.D., Gundelfinger E.D., Garner C.C. (2013). Bassoon and Piccolo maintain synapse integrity by regulating protein ubiquitination and degradation. EMBO J..

[7] Gundelfinger E.D., Reissner C., Garner C.C. (2015). Role of Bassoon and Piccolo in Assembly and Molecular Organization of the Active Zone. Front. Synaptic Neurosci..

[8] Ivanova D., Dirks A., Montenegro-Venegas C., Schöne C., Altrock W.D., Marini C., Frischknecht R., Schanze D., Zenker M., Gundelfinger E.D. (2015). Synaptic activity controls localization and function of CtBP1 via binding to Bassoon and Piccolo. EMBO J..

[9] Sullivan P.F., Geus E.J.C., Willemsen G., James M.R., Smit J.H., Zandbelt T., Arolt V., Baune B.T., Blackwood D., Cichon S. (2009). Genome-wide association for major depressive disorder: A possible role for the presynaptic protein piccolo. Mol. Psychiatry.

[10] Shadrina M., Bondarenko E.A., Slominsky P.A. (2018). Genetics Factors in Major Depression Disease. Front. Psychiatry.

[11] Choi K.H., Higgs B.W., Wendland J.R., Song J., McMahon F.J., Webster M.J. (2011). Gene Expression and Genetic Variation Data Implicate PCLO in Bipolar Disorder. Biol. Psychiatry..

[12] Fujimoto K., Shibasaki T., Yokoi N., Kashima Y., Matsumoto M., Sasaki T., Tajima N., Iwanaga T., Seino S. (2002). Piccolo, a Ca^2+^ sensor in pancreatic beta-cells: Involvement of cAMP-GEFII.Rim2. Piccolo complex in cAMP-dependent exocytosis. J. Biol. Chem..

[13] Ma L., Hanson R.L., Que L.N., Guo Y., Kobes S., Bogardus C., Baier L.J. (2008). PCLO Variants Are Nominally Associated with Early-Onset Type 2 Diabetes and Insulin Resistance in Pima Indians. Diabetes.

[14] Brasil Indígena. https://indigenas.ibge.gov.br/images/pdf/indigenas/folder_indigenas_web.pdf.

[15] Cuautle-Rodríguez P., Llerena A., Molina-Guarneros J. (2014). Present status and perspective of pharmacogenetics in Mexico. Drug Metab. Drug Interact..

[16] Pinto P., Salgado C., Santos N.P.C., Santos S., Ribeiro-dos-Santos Â. (2015). Influence of Genetic Ancestry on INDEL Markers of *NFKβ1*, *CASP8*, *PAR1*, *IL4* and *CYP19A1* Genes in Leprosy Patients. PLoS Negl. Trop. Dis..

[17] Carvalho D.C., Wanderley A.V., Dos Santos A.M.R., Moreira F.C., de Sá R.B.A., Fernandes M.R., Modesto A.A.C., de Souza T.P., Cohen-Paes A., Leitão L.P.C. (2020). Characterization of pharmacogenetic markers related to Acute Lymphoblastic Leukemia toxicity in Amazonian native Americans population. Sci. Rep..

[18] Leal D.F.V.B., Silva M.N.S., Fernandes D.C.R.O., Rodrigues J.C.G., Barros M.C.C., Pinto P.D.C., Pastana L.F., Silva C.A., Fernandes M.R., Assumpção P.P. (2020). Amerindian genetic ancestry as a risk factor for tuberculosis in an amazonian population. PLoS ONE.

[19] Sambrook J., Green M.R. (2012). Molecular Cloning: A Laboratory Manual.

[20] Benjamini Y., Hochberg Y. (1995). Controlling the False Discovery Rate: A Practical and Powerful Approach to Multiple Testing. J. R. Stat. Soc..

[21] Cases-Langhoff C., Voss B., Garner A.M., Appeltauer U., Takei K., Kindler S., Veh R.W., De Camilli P., Gundelfinger E.D., Garner C.C. (1996). Piccolo, a novel 420 kDa protein associated with the presynaptic cytomatrix. Eur. J. Cell Biol..

[22] Rubin G.M., Yandell M.D., Wortman J.R., Gabor G.L., Nelson C.R., Hariharan I.K., Fortini M.E., Li P.W., Apweiler R., Fleischmann W. (2000). Comparative genomics of the eukaryotes. Science.

[23] Ahmed M.Y., Chioza B.A., Rajab A., Schmitz-Abe K., Al-Khayat A., Al-Turki S., Baple E.L., Patton M.A., Al-Memar A.Y., Hurles M.E. (2015). Loss of PCLO function underlies pontocerebellar hypoplasia type III. Neurology.

[24] Bochdanovits Z., Verhage M., Smit A.B., de Geus E.J., Posthuma D., Boomsma D.I., Penninx B.W., Hoogendijk W.J., Heutink P. (2009). Joint reanalysis of 29 correlated SNPs supports the role of PCLO/Piccolo as a causal risk factor for major depressive disorder. Mol. Psychiatry.

[25] Salzano F.M. (1998). Genetic diversity of South American human populations at the DNA and protein levels. J. Exp. Zool..

[26] Ribeiro-dos-Santos A.M., de Souza J.E., Almeida R., Alencar D.O., Barbosa M.S., Gusmão L., Silva W.A., de Souza S.J., Silva A., Ribeiro-dos-Santos Â. (2013). High-Throughput Sequencing of a South American Amerindian. PLoS ONE.

[27] Rodrigues J.C.G., Fernandes M.R., Guerreiro J.F., da Silva A.L.D.C., Ribeiro-Dos-Santos Â., Santos S., Santos N.P.C.D. (2019). Polymorphisms of ADME-related genes and their implications for drug safety and efficacy in Amazonian Amerindians. Sci. Rep..

[28] Rodrigues J.C.G., Souza T.P., Pastana L.F., Ribeiro Dos Santos A.M., Fernandes M.R., Pinto P., Wanderley A.V., Souza S.J., Kroll J.E., Pereira A.L. (2020). Identification of NUDT15 gene variants in Amazonian Amerindians and admixed individuals from northern Brazil. PLoS ONE.

[29] Dobbin E.A.F., Medeiros J.A.G., Costa M.S.C.R., Rodrigues J.C.G., Guerreiro J.F., Kroll J.E., Souza S.J., Assumpcao P.P., Ribeiro-Dos-Santos A.K.C., Santos S.E. (2021). Identification of Variants (rs11571707, rs144848, and rs11571769] in the *BRCA2* Gene Associated with Hereditary Breast Cancer in Indigenous Populations of the Brazilian Amazon. Genes.

[30] Kittles R.A., Chen W., Panguluri R.K., Ahaghotu C., Jackson A., Adebamowo C.A., Griffin R., Williams T., Ukoli F., Adams-Campbell L. (2002). CYP3A4-V and prostate cancer in African Americans: Causal or confounding association because of population stratification?. Hum. Genet..

[31] Skoglund P., Reich D. (2016). A genomic view of the peopling of the Americas. Curr. Opin. Genet. Dev..

[32] Wang S., Lewis C.M., Jakobsson M., Ramachandran S., Ray N., Bedoya G., Rojas W., Parra M.V., Molina J.A., Gallo C. (2007). Genetic Variation and Population Structure in Native Americans. PLoS Genet..

[33] Santos N.P., Ribeiro-Rodrigues E.M., Ribeiro-Dos-Santos A.K., Pereira R., Gusmão L., Amorim A., Guerreiro J.F., Zago M.A., Matte C., Hutz M.H. (2010). Assessing individual interethnic admixture and population substructure using a 48–insertion-deletion (INSEL) ancestry-informative marker (AIM) panel. Hum. Mutat..

[34] Amador M.A., Cavalcante G.C., Santos N.P., Gusmão L., Guerreiro J.F., Ribeirodos-Santos Â., Santos S. (2016). Distribution of allelic and genotypic frequencies of IL1A, IL4, NFKB1 and PAR1 variants in Native American, African, European and Brazilian populations. BMC Res. Notes.

[35] Cavalli-Sforza L.L., Menozzi P., Piazza A. (1994). The History and Geography of Human Genes.

[36] Jobling M.A., Hurles M.E., Tyler-Smith C. (2004). Human Evolutionary Genetics: Origins, Peoples & Disease.

